# A Holocene landscape dynamic multiproxy reconstruction: How do interactions between fire and insect outbreaks shape an ecosystem over long time scales?

**DOI:** 10.1371/journal.pone.0204316

**Published:** 2018-10-02

**Authors:** Lionel Navarro, Anne-Élizabeth Harvey, Adam Ali, Yves Bergeron, Hubert Morin

**Affiliations:** 1 Département des Sciences fondamentales, Université du Québec à Chicoutimi, Chicoutimi, Québec, Canada; 2 Institut des Sciences de l’Évolution, Montpellier, UMR 5554 CNRS-IRD-Université Montpellier-EPHE, Montpellier, France; 3 Institut de recherche sur les forêts, Université du Québec en Abitibi-Témiscamingue, boul. de l’Université, Rouyn-Noranda, Québec, Canada; Royal Holloway University of London, UNITED KINGDOM

## Abstract

At a multi-millennial scale, various disturbances shape boreal forest stand mosaics and the distribution of species. Despite the importance of such disturbances, there is a lack of studies focused on the long-term dynamics of spruce budworm (*Choristoneura fumiferana* (Clem.)) (SBW) outbreaks and the interaction of insect outbreaks and fire. Here, we combine macrocharcoal and plant macrofossils with a new proxy—lepidopteran scales—to describe the Holocene ecology around a boreal lake. Lepidopteran scales turned out to be a more robust proxy of insect outbreaks than the traditional proxies such as cephalic head capsules and feces. We identified 87 significant peaks in scale abundance over the last 10 000 years. These results indicate that SBW outbreaks were more frequent over the Holocene than suggested by previous studies. Charcoal accumulation rates match the established fire history in eastern Canada: a more fire-prone early and late Holocene and reduced fire frequency during the mid-Holocene. Although on occasion, both fire and insect outbreaks were coeval, our results show a generally inverse relationship between fire frequency and insect outbreaks over the Holocene.

## Introduction

Boreal forests are subjected to various natural disturbances that operate at different spatio-temporal scales [1,2] affecting biodiversity and ecosystem dynamics [3,4]. In Canada, fire and insect outbreaks are the two main disturbances that shape forest stand age and composition [5,6]. Although anthropic disturbances (forest harvesting, hydroelectrical infrastructures, oil and gas extraction, etc.) have had a significant impact on terrestrial ecosystems over the last centuries [7], natural disturbances continue to operate at a pluri-millennial scale [8,9].

Dendrochronological studies, through use of fire scars and reductions in tree ring growth, provide valuable insight into the history of fire and insect outbreaks. However, although dendrochronology offers a high resolution (yearly) reconstruction of disturbances, it is limited by the length of the assembled time series. The scarcity of long-term dendrochronological records limits the assessment of disturbances over millennial time scales [10].

To overcome this limitation, paleoecology, via the analysis of proxies preserved in the sedimentary record, provides an opportunity for reconstructing interactions between organisms and their environment across a longer time scale. Use of micro- and macrocharcoal pieces as proxies of fire frequency and intensity as proven as effective tools for developing a detailed portrait of Holocene fire history in the boreal forest. In contrast, the reconstruction of insect outbreaks remains poorly developed.

Spruce budworm (*Choristoneura fumiferana* (Clem.)) (SBW) is the most important defoliator of conifer stands in eastern North America, causing significant growth reductions and mortality in host trees, damaging extensive areas of forest during outbreaks. Despite the scale and impact of SBW outbreaks during the 20^th^ century, the understanding of long-term variability of SBW has been limited due to the lack of indicators.

Simard et al. [9,11] reconstructed 8 200 years of insect activity using insect macrofossils, such as feces and cephalic head capsules, as well as transversal sections of spruce needle casts (*Lophodermium piceae* (Fckl.))—a black spruce (*Picea mariana* (Mill.) B.S.P.) needle endophyte [12]. At their study site, located in the southeastern portion of the boreal forest in Quebec, they identified two main periods of SBW abundance: ca. 6500 cal yr BP and the 20^th^ century, suggesting that intense SBW outbreaks were relatively rare during the Holocene. The authors also suggested that these two main periods of SBW abundance occurred when fire activity was low, favoring the development of mature host stands, suitable for SBW outbreaks.

A 10 000 years fire history from the same region of Quebec showed that in the early Holocene, fire frequency was higher, favoring fire-adapted species, such as jack pine (*Pinus banksiana* Lamb.) [13]. As climate conditions became wetter, ca. 4500 cal yr BP, the fire return intervals were longer and species being less adaptated to fire, such as balsam fir (*Abies balsamea* (L.) Mill.), became more predominant. These large-scale patterns of vegetation change and fire frequency match the δ^18^O temperature reconstructions for the Holocene [14]. From the North American Pollen Database (NAPD), Viau et al. [15] used over 700 radiocarbon-dated pollen time series to highlight two major periods of warming: the first between 14000 and 8000 cal yr BP and a second from 6000 to 3000 cal yr BP. The cooler periods occurred between 8000 and 6000 cal yr BP and from 3000 cal yr BP to the present. In addition, the authors identified a higher frequency, 1 100-year cycle of ± 0.2 °C during the entire Holocene. The Little Ice Age (1350–1850 AD) and the Medieval Warm Period (1100–1200 AD) [16–18] are the most recent manifestations of this high-frequency variability.

Thus, existing boreal landscapes represent a combined inheritance of natural disturbance history and climate conditions occurring over the Holocene [19,20]. While the interactions between fire activity and climate at different time and space scales are relatively well understood for the boreal forest [21–23], the interactions between insect outbreaks, fire and climate remain uncertain.

In a previous publication [24], we presented a new methodology that overcame the shortage of long-term SBW indicators. We showed that fossil lepidopteran wing scales extracted from lake sediments may act as indicators of SBW abundance. This proxy is well-preserved in sediments due to its inert chitinous composition. It also requires a relatively small sample volume (in comparison to using cephalic head capsules and feces). Lepidopteran wing scales can then be combined with other more traditional proxies to reconstruct a landscape dynamic based on insect outbreaks as well as fire history and the resulting vegetation mosaic.

This study uses this new approach to reconstruct a multi-millennial history of SBW abundance, analyze interactions between SBW abundance and fire and assess the role of SBW on forest compositions in the context of Holocene climate variation. We use lepidopteran wing scales in combination with charcoal and plant macrofossils (e.g. needles, leaves, seeds) to reconstruct, for the first time, a Holocene-scale history of the boreal landscape that includes changes in vegetation, fire frequency, and insect outbreaks.

We hypothesize that lepidopteran scales should be found throughout the entire stratigraphy. Scattered episodes of high scale abundance should correspond to periods of warmer temperatures and lower fire activity which contribute to a good development of the insect populations and a high abundance of mature host trees. Scale abundance should also be correlated with the presence of fir and spruce macrofossils within the sediment record. In a much shorter time scale, higher scale abundance should also increase short-term fire frequency as insect-related defoliation increase the risk of ignition, providing an important quantity of flammable fuel to the forest ground [25].

## Materials and methods

The study was conducted in the university experimental forest and we did not need any permission to sample the sites for this study. The field study did not involve endangered or protected species. This study did not imply vertebrate species.

### Site selection and sediment coring

**Fig 1:**
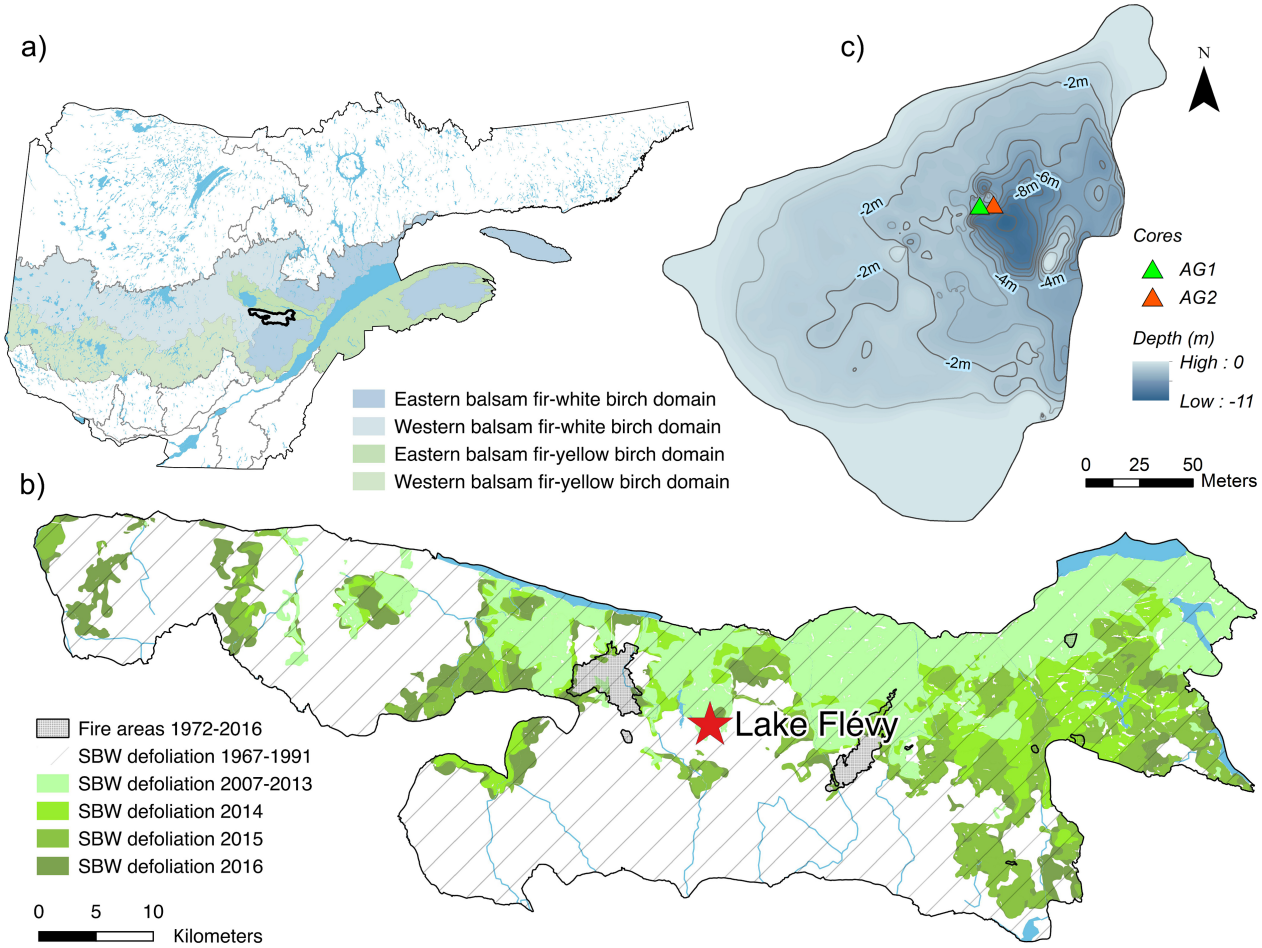
Location of the study site: (a) bioclimatic domains in the south-central portion of Quebec, Canada, (b) a map of historical disturbances surrounding Lake Flévy and (c) a bathymetric map of Lake Flévy showing core sites.

The study area, Lake Flévy (48°13’00”N; 71°12’57”W), is in southeastern Quebec at the interface between the eastern balsam fir-white birch domain and the western balsam fir-white birch domain (Fig 1). The former domain is known to be drier and to has a shorter fire return interval [26]. Lake Flévy is also located in the northern portion of the present-day spruce budworm distribution providing possible insight on the evolution of species distribution under changing climate conditions [27]. The surrounding landscape is characterized by highly sloping, forest-covered hills dominated by spruce-fir stands, particularly on well-drained mesic to hydric locations (hill tops and valley bottoms). Lake Flévy is surrounded by even-aged stands of trembling aspen (*Populus tremuloides* (Michx.)) and mixed even-aged stands of black spruce and aspen. These forest stands originated after an intense fire that occurred in 1922 AD [28] (Fig 1).

SBW has been present in the area for a minimum of 8 240 years [9] and a number of outbreaks have occurred over the last three centuries, becoming more frequent during the 20th century [29]. Aerial surveys indicate that since 2012 AD, SBW-related defoliation has occurred in the resinous stands around the lake reaching severe levels after 2015 AD [30,31] (Fig 1). Regional climate is continental subpolar with a mean annual temperature of 0 °C, mean annual precipitation of 1000 mm and an average growing season of 155 days [32].

The study lake is located in the university experimental forest of Simoncouche and no permission was required in order to sample this site. The field study did not involve endangered or protected species.

The lake was chosen for its small size (2.33 ha) and limited inflow and outflow to the lake, thereby favouring a relatively high sedimentation rate and the retention of a local signal within the sediment record (Fig 1). Two cores were extracted from the deepest portion of the lake (4.8 m) using a Livingstone piston corer. The first core (AG1) was analyzed at the Université du Québec à Montréal (UQAM) for plant macrofossils and charcoal. The second core (AG2) was analyzed at Université du Québec à Chicoutimi (UQAC) for SBW microfossils.

### Sample preparation

**Fig 2:**
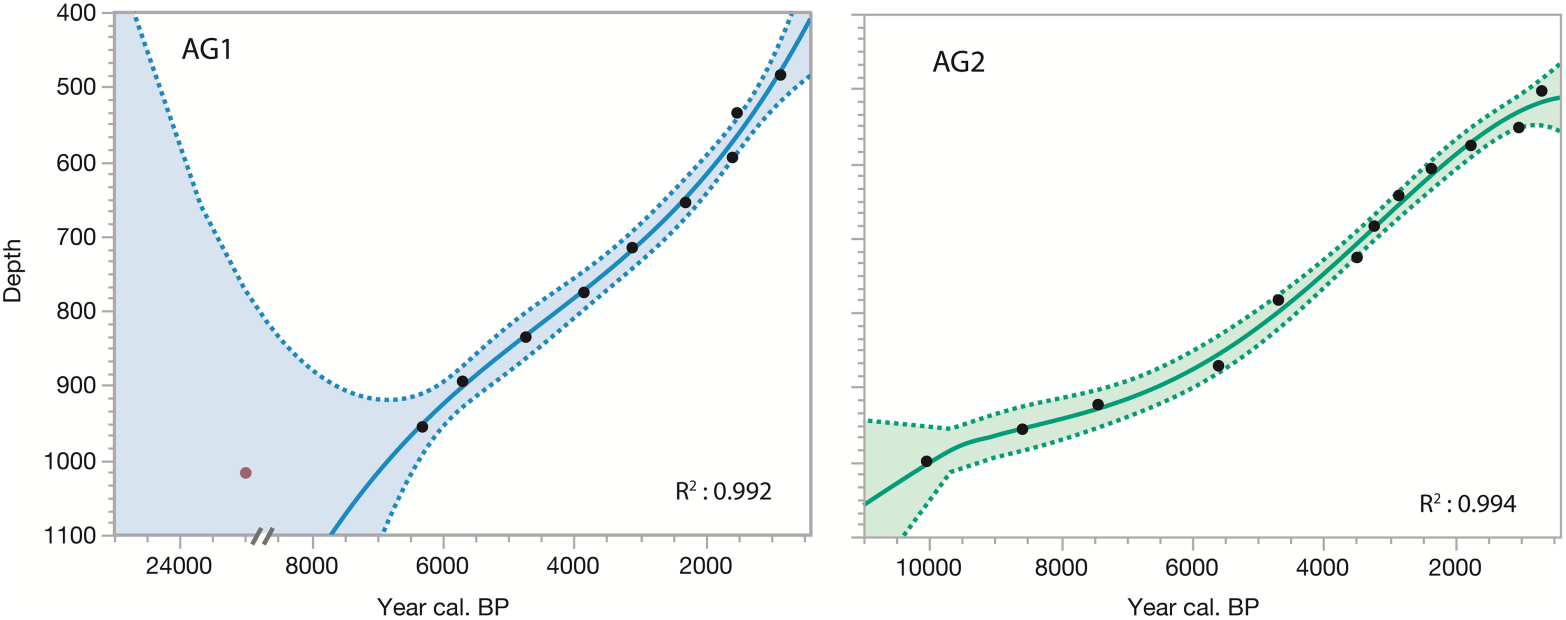
Age-depth model of the sedimentary cores collected at Lake Flévy, Quebec. Error bars represent measurement error. Gray shading of age-depth curve reflects calibration curve error. Sedimentation rates represent a linear interpretation between radiocarbon dates.

The chronological framework for both cores was determined using AMS radiocarbon dating of organic sediments. Samples from AG1 were analyzed at the Beta Analytic Lab in Miami, Florida. Samples from AG2 were prepared in the radiochronology laboratory of Université Laval’s Centre for Northern Studies, then sent to the Keck Carbon Cycle AMS Laboratory, University of California, Irvine. The InCal13 database was used for calibrating ^14^C dates [33]. Sediment accumulation rates were calculated applying a 3^rd^ degree polynomial model using the Clam 2.2 software (Fig 2). All dates are expressed as calibrated years BP (cal yr BP) (Table 1).

**Table 1:** Radiocarbon ages obtained from dating of organic sediment from the sediment cores AG1 and AG2. Calibrated ages determined using the IntCal13 calibration curve [33].

Reference	Site and depth (cm)	Dated material	^14^C age (BP)	±	Calibrated Age Range 2σ (cal Yr BP)	Median (cal yr BP)
	**AG1**					
Beta—401650	482–487	Organic sediment	930	30	787–922	853
Beta—401649	533–537.5	Organic sediment	1620	30	1413–1567	1515
Beta– 401648	593–597	Organic sediment	1680	30	1529–1629	1585
Beta—401647	652.5–658	Organic sediment	2250	30	2157–2265	2299
Beta—401646	712.5–718	Organic sediment	2950	30	3002–3207	3110
Beta—401645	774–777.5	Organic sediment	3550	30	3812–3923	3846
Beta—401644	833–838	Organic sediment	4190	30	4624–4763	4730
Beta—401643	893.5–896	Organic sediment	4970	30	5611–5747	5693
Beta—401642	955–956.5	Organic sediment	5510	30	6276–6353	6304
Beta– 401641*	**1002–1002.5**	**Organic sediment**	**19190**	**80**	**22870–23427**	**23122**
	**AG2**					
ULA-7074	503–504	Organic sediment	725	20	661–688	675
ULA-7075	552–553	Organic sediment	1135	20	969–1081	1024
ULA-7076	576–577	Organic sediment	1810	20	1699–1818	1753
ULA-7077	607–608	Organic sediment	2360	20	2341–2433	2356
ULA-7078	643–644	Organic sediment	2760	15	2791–2882	2851
ULA-7079	684–685	Organic sediment	3030	15	3173–3253	3225
ULA-7080	726–727	Organic sediment	3265	15	3451–3513	3486
ULA-7081	783–784	Organic sediment	4140	15	4581–4725	4683
ULA-7091	871–872	Organic sediment	4850	15	5585–5605	5595
ULA-7092	923–924	Organic sediment	6505	15	7416–7459	7428
ULA-7093	956–957	Organic sediment	7800	15	8547–8600	8579
ULA-7096	999–1000	Organic sediment	8880	20	9981–10156	10037

* Denotes date deemed as an outlier.

Both cores were subsampled at a centimeter-scale resolution. For charcoal extraction, a ca. 1 cm^3^ subsample was collected at each centimeter of AG1. This sediment was deflocculated in a 100 mL, 3% NaP_2_O_4_ solution for 3 h. Each sample was then sieved through a 160 μm mesh to collect charcoal derived from local fires [34], then soaked in a 10% NaOCl solution to bleach the organic matter and help discriminate charcoal fragments.

The remaining AG1 sediment was deflocculated and then sieved through a 160 μm mesh. The recovered >160 μm fraction was transferred to petri dishes. Macrofossil remains, such as needles, twig, seeds, and cone scales, were analyzed using a binocular microscope and identified based on comparisons with reference collections [35–37].

Samples from AG2 were prepared following Navarro et al. [24]. Each sample was dried at 105 °C for 24 h to recover a 0.5 g subsample of dry sediment (± 5 cm^3^). These samples were then heated in a 100 mL 10% potassium hydroxide (KOH) solution at 70 °C for 30 min or until complete deflocculation had occurred. The slurry was then sieved through a 53 μm mesh to retain most scales. We centrifuged the samples at 500 rcf for 10 min in a 10 mL sucrose solution (relative density = 1.24) to remove higher density particles. The centrifuging was repeated three times. After each run, we recovered the supernatant, refilled the vial with the sucrose solution, and centrifuged again. We combined the three supernatants in a 50 mL plastic vial and, to precipitate scales and any remaining particles, centrifuged the combined supernatant at 3900 rcf for 20 min. The final pellet was mounted onto microscope slides for microfossil counting.

### Data handling and identification of peaks

Accumulation rates were calculated using CharAnalysis 1.1 [38]. In order to balance sedimentation rates variations amoung the two cores, each proxy concentration values and deposition times were interpolated to pseudo-annual intervals. The resulting values were then integrated over 5-year intervals and divided by the average deposition time over those intervals [39]. The influx series so obtained (C_int_) allows for core to core comparaision and peak analysis. A stratigraphy was developed using the accumulation rates of each indicator. In Psimpoll 4.27, we used stratigraphically constrained cluster analysis (CONISS) to define three different assemblage zones [40]. Principal component analysis (PCA) was used to identify the main variables that explain the evolution of the ecosystem within these zones.

To extract fire events from the charcoal stratigraphy, a background component (C_back_) was defined using a Lowess smoothing that was robust to outliers and that had a smoothing window of 500 years. C_back_ have been extracted from the interpolated serie of raw data (C_int_) to define a peak serie (C_peak_) as a residual of C_int_-C_back_. A noise component (C_noise_) was calculated for the C_peak_ series using a Gaussian mixture model. A threshold for fire detection (C_fire_) was defined based on the 99^th^ percentile of the C_noise_ distribution. Cfire samples and samples preceeding the Cfire sample that have a >5% probability of being from the same Poisson distribution were discarded. From the fire event dates, we calculated fire frequency over a 500 years bandwidth and smoothed this using a Lowess smoother.

This procedure was also used for insect outbreak detection and frequency. In the same way that a charcoal peak is associated with a fire event, a scale peak is associated by charanalysis with a period of high lepidotera abundance. It is important to note, however, that at this temporal scale, a peak could represent multiple outbreaks. All scales from samples that were associated with a peak were identified so as to confirm a correspondence to a known outbreaking lepidopteran morphotype [24]. ANOVA was used to determine the mean differences between the relative occurrence of each species morphotype, and a Tukey’s range test determined whether the means were significantly different.

## Results

Core AG1 was 519 cm long and was dated to more than 6000 cal yr BP (Fig 2). One date was excluded from the age-depth model as it was considered as outlier (Table 1). Indeed, the radiocarbon date at the bottom of AG1 (Beta– 401641) was probably influenced by the presence of stromatolites in the sampling lake (B. Lapointe, personal communication), probably originating from the lac Mistassinni [41] The sediment accumulation rate was relatively stable across the core varying from 6 to 16 yr·cm^-1^. Core AG2 was 511 cm long and represented almost 10 000 yr of deposition. The sediment accumulation rate of AG2 was more than two times slower than that of AG1 from 10000 to 6000 cal yr BP, then was more similar to that of core AG1 over the last 6000 years (Fig 2).

**Fig 3:**
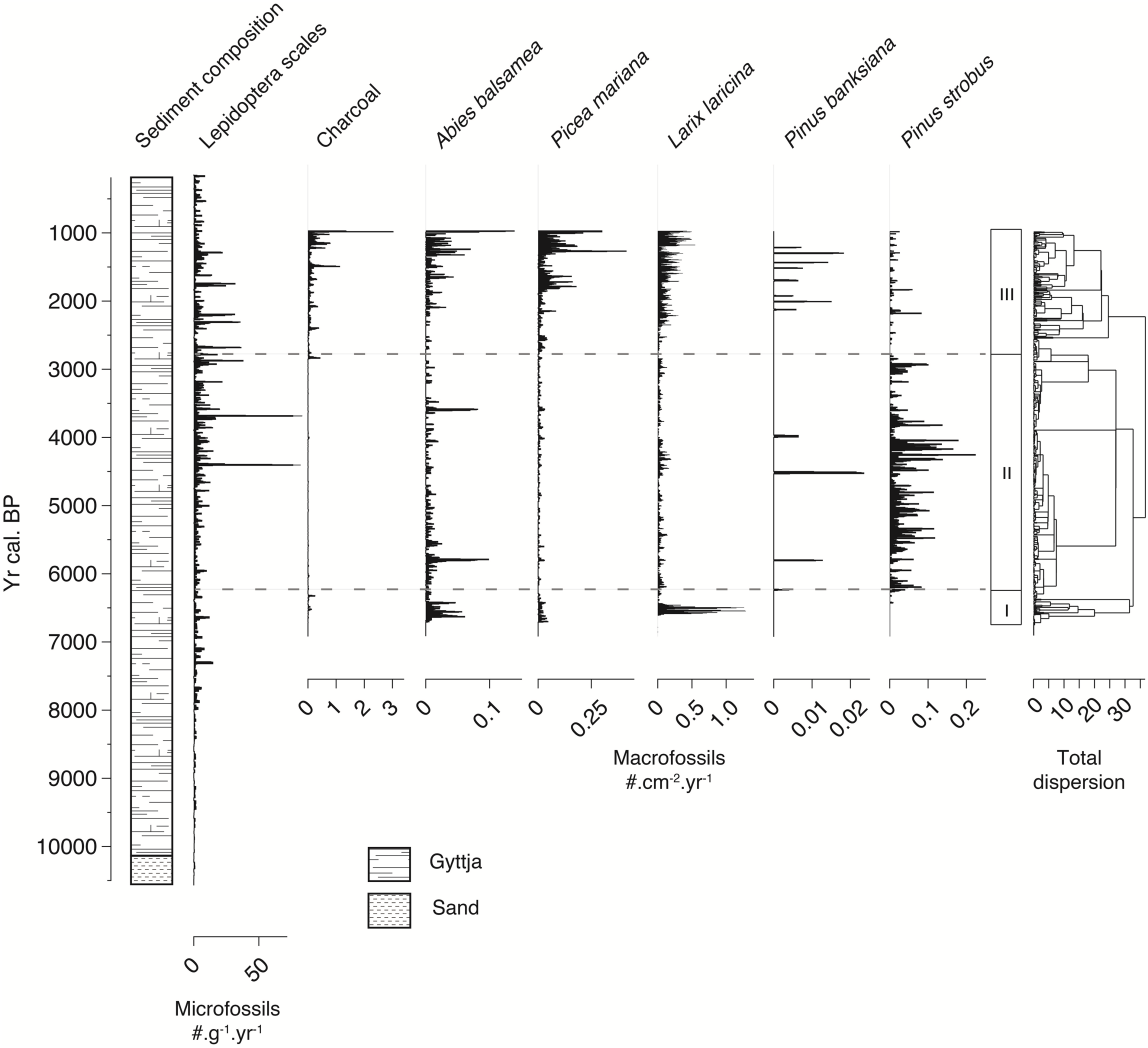
Stratigraphy of accumulation rates for lepidopteran scales (#·g^-1^·yr^-1^), charcoal and plant macrofossils (#·cm^-2^·yr^-1^) from cores recovered from Lake Flévy. Lepidopteran scales were counted from core AG2. Charcoal and plant macrofossils were counted from core AG1.

The two cores consisted essentially of homogenous organic sediment (gyttja) (Fig 3). CONISS identified three distinct assemblage zones along the composite stratigraphy.

Zone I (6700–6250 cal yr BP) defines the earliest section of AG1. This relatively short time interval had marked presence of larch (*Larix laricina* (Du Roi) K.Koch.), balsam fir and, to a lesser extent, black spruce macrofossils. Some minor charcoal peaks were also identified in this zone.

Zone II (6250–2700 cal yr BP) covers a major portion of the core. Charcoal was nearly absent from this zone that was characterized by the predominance of eastern white pine (*Pinus strobus* (L.)), a more thermophile and less fire-adapted species. In this zone, black spruce, larch, and balsam fir were less common than in Zone I despite some major peaks ca. 3600 and 5800 cal yr BP. Some isolated peaks of jack pine (*Pinus banksiana* (Lamb.)) were also observed during this period. This zone was also marked by a high number of lepidopteran scale peaks around 4500, 3600, and 2900 cal yr BP.

Zone III (2700 cal yr BP to 975 cal yr BP) represents the most recent portion of the lake record. It includes a subzone between 2700 and 2050 cal yr BP representing a transition between zones II and III. In the subzone, every indicator was nearly absent except for lepidopteran scales and some black spruce macrofossils. Between 2050 and 975 cal yr BP, the landscape returned to an early Holocene similar stage characterized by a higher proportion of boreal taxa such as balsam fir, black spruce and larch. Two high magnitude peaks of lepidopteran scales were observed around 1300, 1700 and 2300 cal yr BP. Charcoal, balsam fir, and black spruce macrofossils showed a delay following those lepidopteran peaks. Some remains of jack pine were also identified between 1000 and 2000 cal yr BP, however their abundance was very low relative to the other taxa.

**Fig 4:**
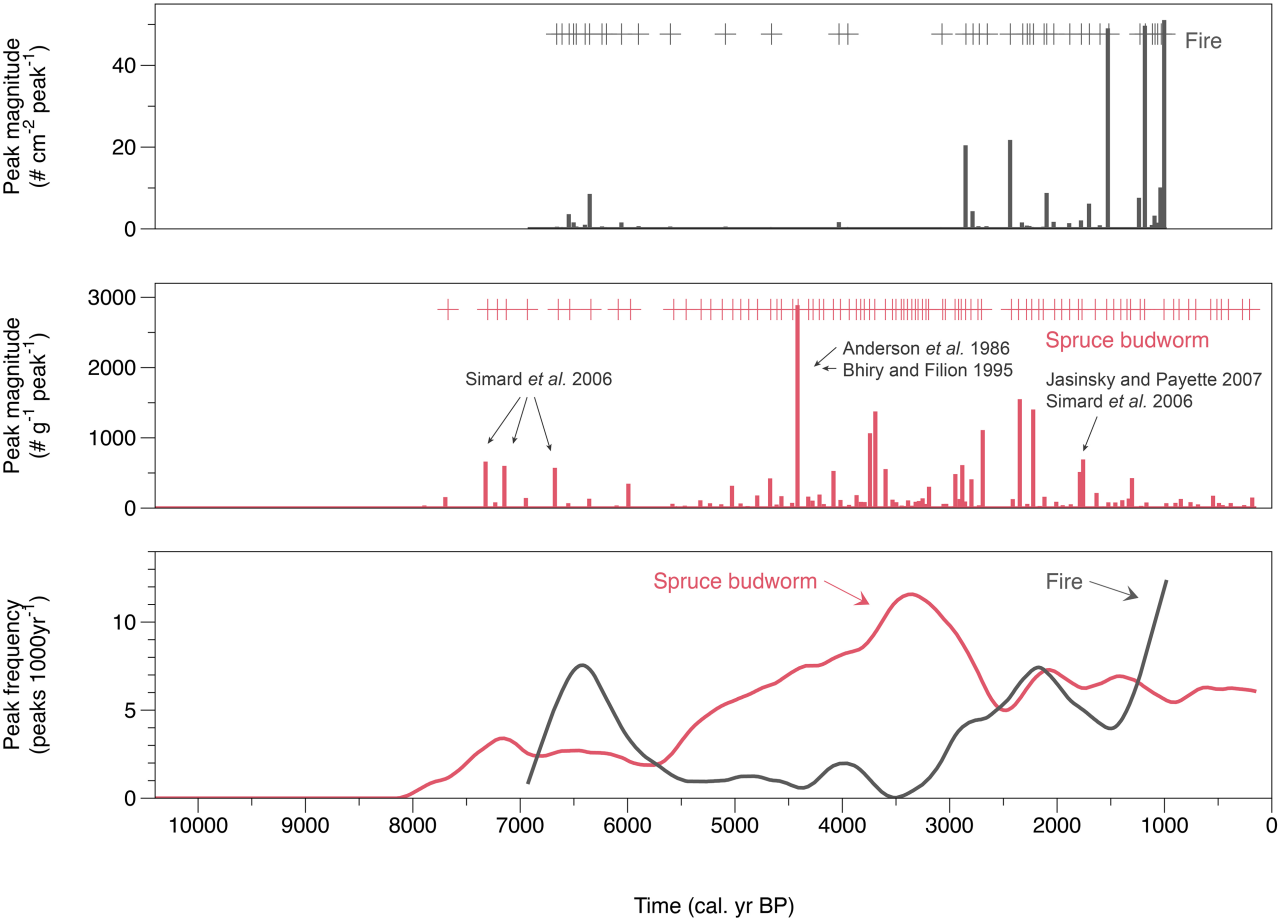
Magnitude of fire (a) and Lepidopteran scale (b) peaks (#·cm^-2^·peak^-1^). Each peak exceed the 99th percentile threshold of the residual of C_int_-C_back_ (c) The frequency of fire and SBW (peaks·1000yr^-1^).

**Fig 5:**
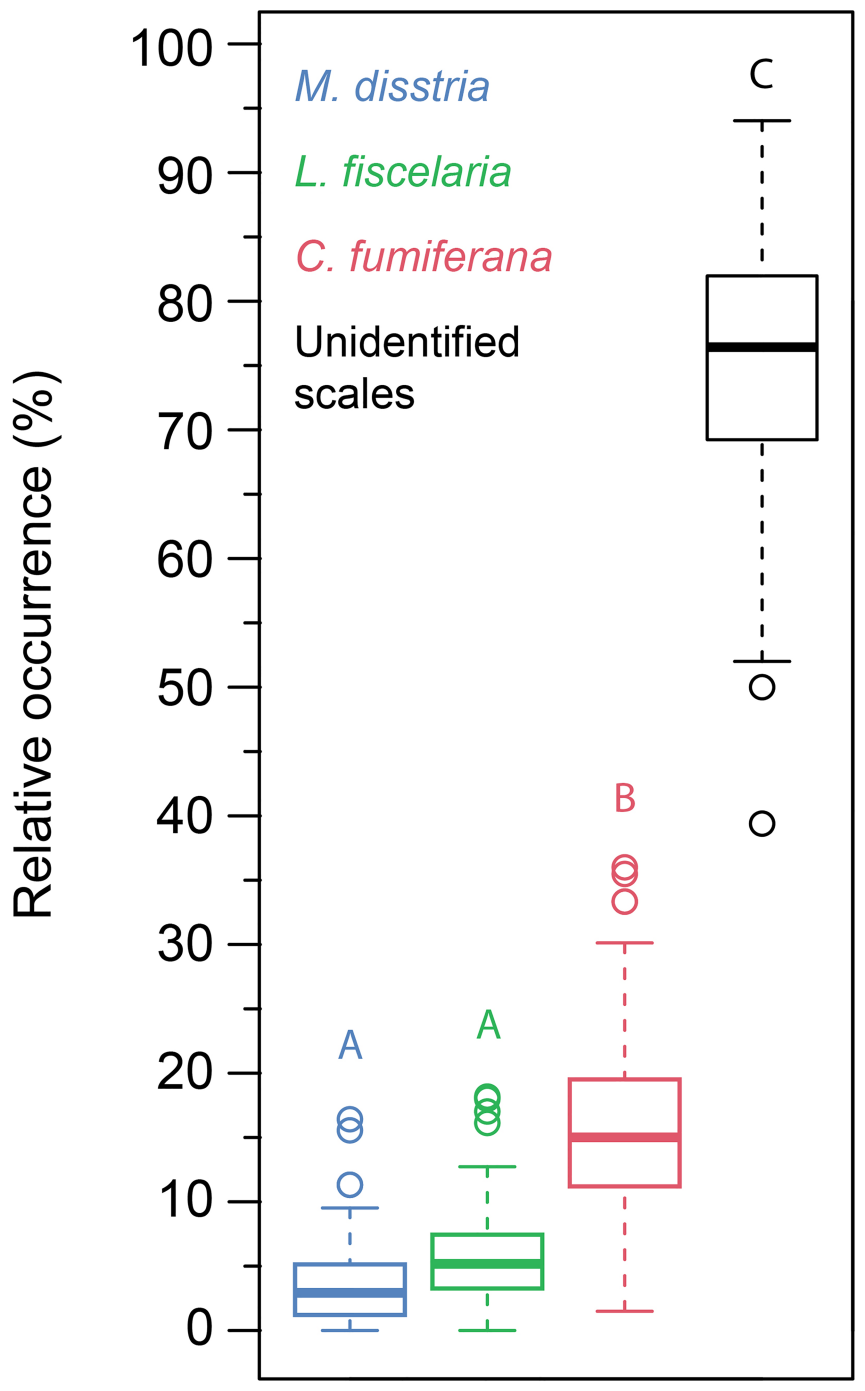
Identification of lepidopteran species identified from the 68 scale peaks from AG2. Malacosoma disstria and Lambdina fiscelaria occurrences were not significantly different (p = 0.28) from each other. Choristoneura fumiferana was significantly more represented than the other outbreak species (p < 0.0001) and the relative abundance of unidentified scales was significantly higher than the relative abundance of identified scales (p < 0.0001).

A total of 87 lepidopteran scale peaks and 41 charcoal peaks were identified from both cores (Fig 4). Although not all scales from these peaks could be identified based on the morphotypes described in Navarro et al. [24], 62% of the identified scales corresponded to a SBW morphotype. The ANOVA confirmed that *Choristoneura fumiferana was significantly more represented than the other outbreak species (p < 0*.*0001)*. Morphotypes of the forest tent caterpillar (*Malacosoma disstria* (Hübner)) and hemlock looper (*Lambdina fiscelaria* (Guénée)) were less frequently identified (Fig 5) and their relative occurence *were not significantly different (p = 0*.*28) from each other*. Identification based solely on shape morphotype prevented us from identifying the majority of those scales (77%) extracted from the peaks as many either were broken or folded.

The frequency of scale peaks was greater than that of charcoal between 5500 and 2500 cal yr BP reaching a frequency of 13 peaks per 1000 years at ca. 3200 cal yr BP. Between 1500 and 1000 cal yr BP there was a reversal of this trend with a fire event rate reaching a maximum frequency of 13 peaks per 1000 years.

**Fig 6:**
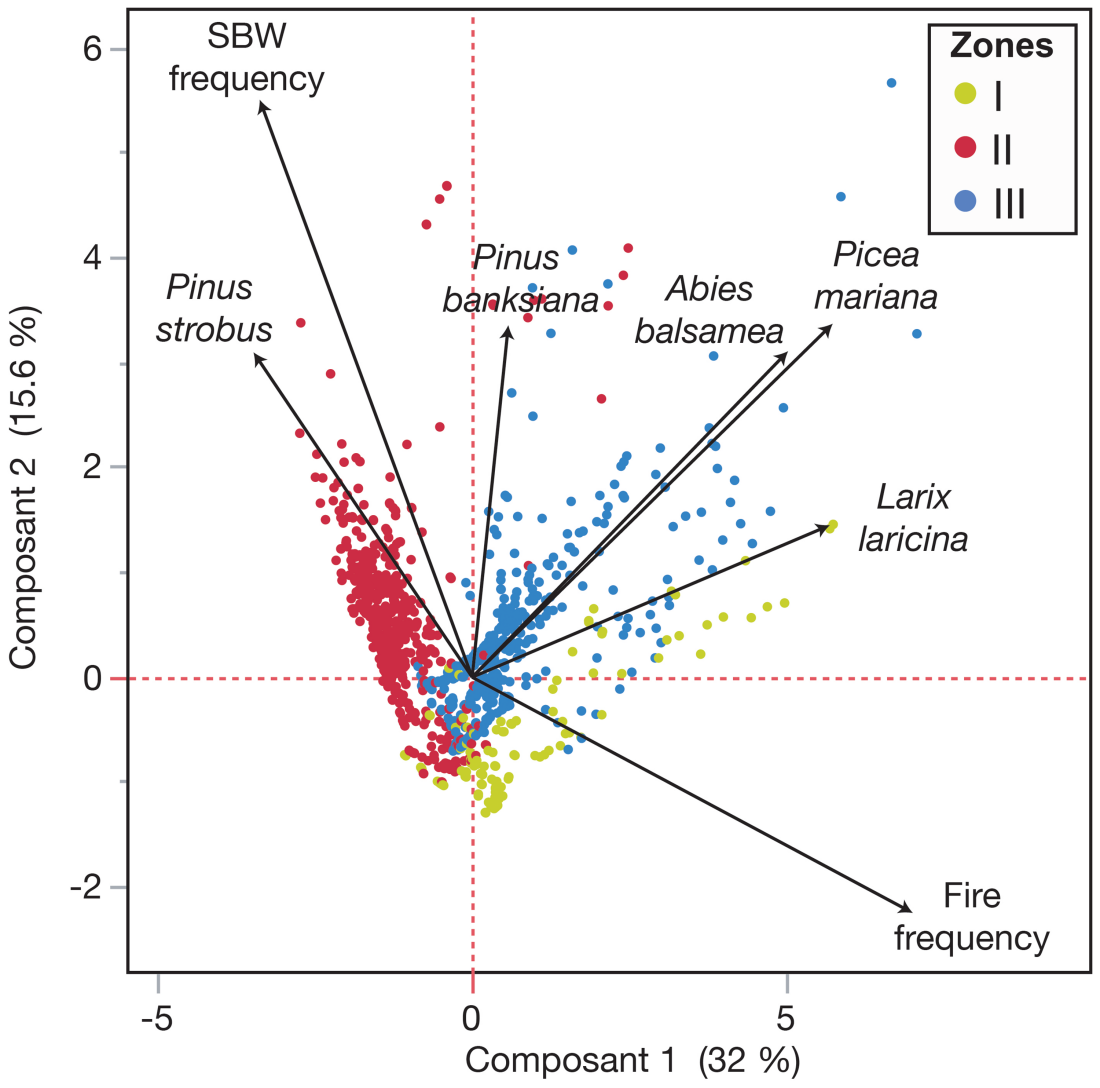
Principal component analysis (PCA) biplot of the mean scores along the first two components. The components were calculated using the different proxy indicators recorded from the two sediment cores recovered from Lake Flévy. Colors were assigned to each CONISS-defined zone: Zone I (early Holocene–6250 cal yr BP; yellow), Zone II (6250–2700 cal yr BP; red) and Zone III (2700 cal yr BP to the present; blue).

The first two axes of the PCA explained 47.6% of the total variation, 32% represented by Axis 1 and 15.6% represented by Axis 2 (Fig 6). The y axis separates Zone 2, mainly influenced by the SBW outbreak frequency and eastern white pine macrofossil abundance, from zones I and III that were more influenced by fire frequency, black spruce and larch macrofossil abundance. Zone I samples were less dispersed than Zone III samples reflecting the higher impact of larch macrofossils. The opposing directions of SBW outbreak frequency and balsam fir macrofossils along with the opposing relationship between fire frequency and eastern white pine macrofossils indicate that these variables varied inversely.

## Discussion

### Fire history and climate

The charcoal accumulation rates from Lake Flévy match the known Holocene fire history in eastern Canada [8,21–23]: a more fire-prone early and late Holocene separated by a reduced fire regime—relative to the present—during the mid Holocene. The Holocene fire history recorded in Lake Flévy tracks the δ^18^O-based, southern Ontario postglacial precipitation reconstruction of Edwards et al. [14] as well as different paleofire regime modelisations from eastern Quebec [21,42,43]. Post- and neoglacial periods were cold and dry leading to a higher fire frequency and a dominance of more fire-adapted trees, such as *Picea mariana*, that are resilient to cooler conditions and drought [44]. Although the mid Holocene was warmer [15], fire activity was very low, likely explained by a high relative humidity during this period [14] and the presence of a more stable air mass leading to less frequent drought events [45].

### SBW outbreaks over the Holocene

Based on our dataset, SBW outbreaks are not a rare phenomenon at the Holocene-scale which is in contradiction with our first hypothesis. Multiple peaks of scale abundance were identified downcore. Some have already been previously noted in a mire of the same area (<10km) using faecal macrorests, including outbreaks ca., 1930, 4500, 6650, 7180 and 7560 cal yr BP [9,12,46,47]. However, the novel use of lepidopteran scales also identified multiple, previously unobserved, periods of high lepidopteran abundance such as events at 2500, 2930, 3110, and 3850 cal yr BP.

The scale peak at 4500 cal yr BP has been linked to a possible combined action from SBW and hemlock looper across northern Maine and southern Quebec [9,46,47]. Based on our identification criteria, outbreaks observed in our study site were not associated with hemlock looper. However, given the morphological similarity of the two species’ scales, the exceptional quantity of microfossils recovered for this period and the unusual recent hemlock looper activity in the Laurentian Wildlife Reserve (in which Lake Flévy is situated) [48], we cannot completely exclude the possibility of the presence of both defoliators in the area.

The high number of scale peaks identified in the Lake Flévy sediment record allows us to propose the first detailed Holocene-scale SBW outbreak record. The frequency of outbreaks varies over time with periods having a few outbreaks events (10000–6000 cal yr BP; 2500–1000 cal yr BP) and periods marked by very frequent events (5500–2500 cal yr BP).

At a finer temporal scale, some lepidopteran outbreaks (1300, 1800, 4650 cal yr BP) seem to be associated with lagged charcoal, *Abies balsamea* and *Picea mariana* macrofossil peaks (Figs 3 and 5). This suggests a direct (and probably combined) influence of these disturbances affecting the input of plant macrofossils into the sediment record.

### Fire-insect interactions

Previous studies [49,50] have shown that SBW outbreaks can enhance fire activity over the short term, providing a 5 to 10 yr window of opportunity during which “ladder fuel” builds up due to crown breakage and windthrow of the killed and damaged trees [49,50]. Following this SBW outbreak-related window, humidity rises and the dead wood generated by the outbreak begins to rot [51].

Using a 300 years model of disturbances interactions, James *et al*. (2011) argue that this ephemeral increase in fire risk due to budworm activity rarely leads to consecutive disturbances at longer timescales [25]. SBW is known to be a cyclic, age-dependent, selective disturbance whereas fire ignition requires a more random event such as a lightning stike. Thus, there is less chance of an immediate succession between these two disturbances. In fact, the two disturbances showed an inverse relationship over the long-term (Fig 6) suggesting an effect of competition for limited resources (mature trees). When fire frequency is low, trees can grow fully and forests can reach a mature stage that enhances SBW activity. On the other hand, when fire frequency increases, there is less time for mature stands to become established, thereby limiting SBW activity.

## Conclusion

Our results constitute an important starting point for combining and understanding the long-term interactions between natural disturbances, such as fire and SBW outbreaks, in the boreal forest. This approach provides valuable insight into long-term forest dynamics and may reveal spatial heterogeneity in disturbances (and their interactions). Recognizing these patterns would favor the development of different types of future sustainable management policies depending on the region. For example, this long-term approach could be applied to reconstructing western spruce budworm (*Choristoneura occidentalis* (Free.)) or mountain pine beetle (*Dendroctonus ponderosae* (Hopk.)) infestations in western Canada and United States and determining the interactions between outbreaks, fire, and climate in these regions. In a context of global change, it is critical to understand the long-term dynamics of the boreal ecosystem and combined role of multiple disturbances.

## References

[1] Turner MG, Romme W, Gardner RH, O’Neill R, Kratz T. A revised concept of landscape equilibrium: Disturbance and stability on scaled landscapes. Landsc Ecol. 1993;8: 213–227.

[2] Turner MG. Disturbance and landscape dynamics in a changing world. Ecology. 2010;91: 2833–2849. 10.1890/10-0097.1 21058545

[3] Cardinale BJ, Palmer MA, Ives AR, Brooks SS. Diversity-productivity relationships in streams vary as a function of the natural disturbance regime. Ecology. 2005;86: 716–726. 10.1890/03-0727

[4] Arseneault D, Sirois L. The millennial dynamics of a boreal forest stand from buried trees. J Ecol. 2004;92: 490–504. 10.1111/j.0022-0477.2004.00887.x

[5] Gauthier S, Leduc A, Harvey B, Bergeron Y, Drapeau P. Les perturbations naturelles et la diversité écosystémique. Le Nat Can. 2001;125: 10–17.

[6] Jardon Y, Morin H, Dutilleul P. Périodicité et synchronisme des épidémies de la tordeuse des bourgeons de l’épinette au Québec. Can J For Res. 2003;33: 1947–1961. 10.1139/x03-108

[7] Schindler DW, Lee PG. Comprehensive conservation planning to protect biodiversity and ecosystem services in Canadian boreal regions under a warming climate and increasing exploitation. Biol Conserv. Elsevier Ltd; 2010;143: 1571–1586. 10.1016/j.biocon.2010.04.003

[8] Power MJ, Marlon J, Ortiz N, Bartlein PJ, Harrison SP, Mayle FE, et al Changes in fire regimes since the Last Glacial Maximum: an assessment based on a global synthesis and analysis of charcoal data. Clim Dyn. 2008;30: 887–907. 10.1007/s00382-007-0334-x

[9] Simard I, Morin H, Lavoie C. A millennial-scale reconstruction of spruce budworm abundance in Saguenay, Québec, Canada. The Holocene. 2006;16: 31–37. 10.1191/0959683606hl904rp

[10] Davis RB, Hoskins BR. A new parameter for paleoecological reconstruction: head capsules of forest-tree defoliator microlepidopterans in lake sediment. Abstracts and Program of the 6th Biennial Meeting of the American Quaternary Association, August 18–20. Orono, Maine: University of Maine; 1980. p. 21.

[11] Simard I. Dynamique des épidémies de la tordeuse des bourgeons de l’épinette durant l’holocène. Université du Québec à Chicoutimi. 2011.

[12] Jasinski JPP, Payette S. Holocene occurrence of Lophodermium piceae, a black spruce needle endophyte and possible paleoindicator of boreal forest health. Quat Res. 2007;67: 50–56. 10.1016/j.yqres.2006.07.008

[13] Couillard P, Payette S, Grondin P. Long-term impact of fire on high-altitude balsam fir (Abies balsamea) forests in south-central Quebec deduced from soil charcoal. Can J For Res. 2013;43: 188–199. 10.1139/cjfr-2012-0414

[14] Edwards TWD, Wolfe BB, Macdonald GM. Influence of changing atmospheric circulation on precipitation δ18 O–temperature relations in Canada during the Holocene. Quat Res. 1996;46: 211–218. 10.1006/qres.1996.0061

[15] Viau AE, Gajewski K, Sawada MC, Fines P. Millennial-scale temperature variations in North America during the Holocene. J Geophys Res Atmos. 2006;111: 1–12. 10.1029/2005JD006031

[16] Moberg A, Sonechkin DM, Holmgren K, Datsenko NM, Karlén W, Lauritzen S-E. Highly variable Northern Hemisphere temperatures reconstructed from low- and high-resolution proxy data. Nature. 2005;433: 613–617. 10.1038/nature03265 15703742

[17] El Bilali H, Patterson RT, Prokoph A. A Holocene paleoclimate reconstruction for eastern Canada based on δ18O cellulose of *Sphagnum* mosses from Mer Bleue Bog. The Holocene. 2013;23: 1260–1271. 10.1177/0959683613484617

[18] Wanner H, Beer J, Bütikofer J, Crowley TJ, Cubasch U, Flückiger J, et al Mid- to Late Holocene climate change: an overview. Quat Sci Rev. 2008;27: 1791–1828. 10.1016/j.quascirev.2008.06.013

[19] Grondin P, Gauthier S, Borcard D, Bergeron Y, Noël J. A new approach to ecological land classification for the Canadian boreal forest that integrates disturbances. Landsc Ecol. 2014;29: 1–16. 10.1007/s10980-013-9961-2

[20] Asselin M, Grondin P, Lavoie M, Fréchette B. Fires of the last millennium led to landscapes dominated by early successional species in Québec’s clay belt boreal forest, Canada. Forests. 2016;7: 18 10.3390/f7090205

[21] Ali AA, Blarquez O, Girardin MP, Hely C, Tinquaut F, El Guellab A, et al Control of the multimillennial wildfire size in boreal North America by spring climatic conditions. Proc Natl Acad Sci. 2012;109: 20966–20970. 10.1073/pnas.1203467109 23213207 PMC3529026

[22] Blarquez O, Ali AA, Girardin MP, Grondin P, Fréchette B, Bergeron Y, et al Regional paleofire regimes affected by non-uniform climate, vegetation and human drivers. Sci Rep. Nature Publishing Group; 2015;5: 1–13. 10.1038/srep13356 26330162 PMC4557068

[23] Remy CC, Lavoie M, Girardin MP, Hély C, Bergeron Y, Grondin P, et al Wildfire size alters long-term vegetation trajectories in boreal forests of eastern North America. J Biogeogr. 2017; 1268–1279. 10.1111/jbi.12921

[24] Navarro L, Harvey A-É, Morin H. Lepidoptera wing scales: a new paleoecological indicator for reconstructing spruce budworm abundance. Can J For Res. 2017; Forthcoming.

[25] James PMA, Fortin M-J, Sturtevant BR, Fall A, Kneeshaw D. Modelling spatial interactions among fire, spruce budworm, and logging in the boreal forest. Ecosystems. 2011;14: 60–75. 10.1007/s10021-010-9395-5

[26] Bouchard M, Pothier D, Gauthier S. Fire return intervals and tree species succession in the North Shore region of eastern Quebec. Can J For Res. 2008;38: 1621–1633. 10.1139/X07-201

[27] Régnière J, St-Amant R, Duval P. Predicting insect distributions under climate change from physiological responses: spruce budworm as an example. Biol Invasions. 2012;14: 1571–1586. 10.1007/s10530-010-9918-1

[28] Gagnon R. Maintient après feu de limites abruptes entre des peuplements d’épinette noire (Picea mariana) et des formations de feuillus intolérants (Populus tremoloides et Betula papyrifera) dans la region du Saguenay-Lac-Saint-Jean (Québec). Nat Can. 1989;116: 117–124.

[29] Blais JR. Trends in the frequency, extent, and severity of spruce budworm outbreaks in eastern Canada. Can J For Res. 1983;13: 539–547. 10.1139/x83-079

[30] Ministère des Ressources Naturelles et de la Faune. Aires infestées par la tordeuse des bourgeons de l’épinette au Québec en 2012. Québec; 2012.

[31] Ministère des Forêts de la Faune et des Parcs. Aires infestées par la tordeuse des bourgeons de l’épinette au Québec en 2015. Québec; 2015.

[32] Blouin J, Berger J-P, Landry Y, Saucier J-P. Guide de reconnaissance des types écologiques des régions écologiques 5b—Coteaux du réservoir Gouin, 5c—Collines du haut Saint-Maurice et 5d—Collines ceinturant le lac Saint-Jean. Ministère des Ressources Naturelles et de la Faune, editor. Gouvernement du Québec; 2008.

[33] Reimer PJ, Bard E, Bayliss A, Beck JW, Blackwell PG, Ramsey CB, et al IntCal13 and Marine13 radiocarbon age calibration curves 0–50,000 years cal BP. Radiocarbon. 2013;55: 1869–1887. 10.2458/azu_js_rc.55.16947

[34] Higuera PE, Peters ME, Brubaker LB, Gavin DG. Understanding the origin and analysis of sediment-charcoal records with a simulation model. Quat Sci Rev. 2007;26: 1790–1809. 10.1016/j.quascirev.2007.03.010

[35] Young JA, Young CG. Seeds of woody plants in North America. Fairfield, Ohio: Timber Press; 1992.

[36] Crow G, Hellquist CB. Aquatic and wetland plants of northeastern North America, Volume I: A revised and enlarged edition of Norman C. Fassett’s A manual of aquatic plants, Volume I: pteridophytes, gymnosperms, and angiosperms: dicotyledons. Bibliovault OAI Repository, the University of Chicago Press. 2000.

[37] Crow G, Hellquist CB. Aquatic and wetland plants of northeastern North America, Volume II: a revised and enlarged edition of Norman C. Fassett’s A manual of aquatic plants, Volume II: angiosperms: monocotyledons. Bibliovault OAI Repository, the University of Chicago Press. 2000.

[38] Higuera PE, Brubaker LB, Anderson PM, Hu FS, Brown TA. Vegetation mediated the impacts of postglacial climate change on fire regimes in the south-central Brooks Range, Alaska. Ecol Monogr. 2009;79: 201–219. 10.1890/07-2019.1

[39] Long CJ, Whitlock C, Bartlein PJ, Millspaugh SH. A 9000-year fire history from the Oregon Coast Range, based on a high-resolution charcoal study. Can J For Res. 1998;28: 774–787.

[40] Bennett KD. Psimpoll 4.26: C program for plotting pollen diagrams and analysing pollen data. Belfast: Queen’s University of Belfast; 2007.

[41] Dionne J-C. Blocs de dolomie à stromatolites sur les rives de l’estuaire du Saint-Laurent, Québec. Géographie Phys Quat. 1986;40: 93 10.7202/032626ar

[42] Girardin MP, Ali AA, Carcaillet C, Blarquez O, Hély C, Terrier A, et al Vegetation limits the impact of a warm climate on boreal wildfires. New Phytol. 2013;199: 1001–1011. 10.1111/nph.12322 23691916

[43] Hély C, Girardin MP, Ali AA, Carcaillet C, Brewer S, Bergeron Y. Eastern boreal North American wildfire risk of the past 7000 years: A model-data comparison. Geophys Res Lett. 2010;37: 1–6. 10.1029/2010GL043706

[44] Belien E, Rossi S, Morin H, Deslauriers A. High-resolution analysis of stem radius variations in black spruce [*Picea mariana* (Mill.) BSP] subjected to rain exclusion for three summers. Trees—Struct Funct. 2014;28: 1257–1265. 10.1007/s00468-014-1011-4

[45] Carcaillet C, Richard PJH. Holocene changes in seasonal precipitation highlighted by fire incidence in eastern Canada. Clim Dyn. 2000;16: 549–559. 10.1007/s003820000062

[46] Bhiry N, Filion L. Mid-Holocene hemlock decline in eastern North America linked with phytophagous insect activity. Quat Res. 1996;45: 312–320. 10.1006/qres.1996.0032

[47] Anderson RS, Davis RB, Miller NG, Stuckenrath R. History of late- and post-glacial vegetation and disturbance around Upper South Branch Pond, northern Maine. Can J Bot. 1986;64: 1977–1986. 10.1139/b86-262

[48] Ministère des Ressources Naturelles et de la Faune. Aires infestées par l’arpenteuse de la pruche au Québec en 2012. Québec; 2012.

[49] Stocks BJ. Fire potential in the spruce budworm-damaged forests of Ontario. For Chron. 1987;63: 8–14. 10.5558/tfc63008-1

[50] Fleming RA, Candau J-N, McAlpine RS. Landscape-scale analysis of interactions between insect defoliation and forest fire in central Canada. Clim Change. 2002;55: 251–272. 10.1023/A:1020299422491

[51] James PMA, Robert L-E, Wotton BM, Martell DL, Fleming RA. Lagged cumulative spruce budworm defoliation affects the risk of fire ignition in Ontario, Canada. Ecol Appl. 2017;27: 532–544. 10.1002/eap.1463 27809401

